# Impact of occupational pesticide exposure on the human gut microbiome

**DOI:** 10.3389/fmicb.2023.1223120

**Published:** 2023-08-10

**Authors:** Milla F. Brandao Gois, Asier Fernández-Pato, Anke Huss, Ranko Gacesa, Cisca Wijmenga, Rinse K. Weersma, Jingyuan Fu, Roel C. H. Vermeulen, Alexandra Zhernakova, Virissa C. Lenters, Alexander Kurilshikov

**Affiliations:** ^1^Department of Genetics and Department of Gastroenterology and Hepatology University of Groningen, University Medical Center Groningen, Groningen, Netherlands; ^2^Department of Population Health Sciences, Institute for Risk Assessment Sciences (IRAS), Utrecht University, Utrecht, Netherlands; ^3^Department of Gastroenterology and Hepatology, University of Groningen, University Medical Center Groningen, Groningen, Netherlands; ^4^Department of Pediatrics, University of Groningen, University Medical Center Groningen, Groningen, Netherlands

**Keywords:** gut microbiome, pesticide exposure, exposome, occupational exposure, microbiome

## Abstract

The rising use of pesticides in modern agriculture has led to a shift in disease burden in which exposure to these chemicals plays an increasingly important role. The human gut microbiome, which is partially responsible for the biotransformation of xenobiotics, is also known to promote biotransformation of environmental pollutants. Understanding the effects of occupational pesticide exposure on the gut microbiome can thus provide valuable insights into the mechanisms underlying the impact of pesticide exposure on health. Here we investigate the impact of occupational pesticide exposure on human gut microbiome composition in 7198 participants from the Dutch Microbiome Project of the Lifelines Study. We used job-exposure matrices in combination with occupational codes to retrieve categorical and cumulative estimates of occupational exposures to general pesticides, herbicides, insecticides and fungicides. Approximately 4% of our cohort was occupationally exposed to at least one class of pesticides, with predominant exposure to multiple pesticide classes. Most participants reported long-term employment, suggesting a cumulative profile of exposure. We demonstrate that contact with insecticides, fungicides and a general “all pesticides” class was consistently associated with changes in the gut microbiome, showing significant associations with decreased alpha diversity and a differing beta diversity. We also report changes in the abundance of 39 different bacterial taxa upon exposure to the different pesticide classes included in this study. Together, the extent of statistically relevant associations between gut microbial changes and pesticide exposure in our findings highlights the impact of these compounds on the human gut microbiome.

## 1. Introduction

The concept of the “exposome,” conceived in 2005, seeks to better understand the effects of lifelong environmental exposures in health and disease (Wild, 2005). The exposome encompasses a complex network of factors, including environmental, chemical, behavioral and lifestyle factors (Landrigan et al., 2018; Daiber et al., 2019; Ohlander et al., 2020). Exploring the associations of these exposome components with health outcomes can help identify relevant non-genomic influences on health risks and disease burden (Daiber et al., 2019). Increasing environmental pollution and growing use of pesticides in agriculture have resulted in a shift in the modern burden of diseases, where these elements may have a wide range of health impacts (De Jong et al., 2014; Cosselman et al., 2015; Kim et al., 2017; Stingone et al., 2017; Daiber et al., 2019; Niedzwiecki et al., 2019; Ohlander et al., 2020; Vermeulen et al., 2020).

As pesticides are deliberately designed to be poisonous to certain organisms, unintended exposure to these substances can be potentially hazardous to humans and wildlife (Kim et al., 2017). Currently, biomonitoring data indicates that most of the world population is exposed to these chemicals at some level due to the wide range and large amounts of pesticides in use (Blair et al., 2015; Kim et al., 2017; Andersen et al., 2022; Grau et al., 2022; Nomura et al., 2022; Ospina et al., 2022; Stanfield et al., 2022; Govarts et al., 2023). The unintended encounter with these compounds often occurs via residues present in food, liquids or air (Blair et al., 2015; Figueiredo et al., 2021; Buekers et al., 2022; Connolly et al., 2022; Grau et al., 2022), but employment settings in particular are linked to higher levels of exposure, and workers are likely to be repetitively exposed (Blair et al., 2015; Curl et al., 2020; Peters et al., 2021). As a consequence, there is a body of evidence for association of pesticide exposure with increased risk for several diseases, including metabolic and immune disorders, cancers and neurological outcomes (Blair et al., 2015; Cosselman et al., 2015; Parks et al., 2019; Curl et al., 2020; Fuhrimann et al., 2021).

In parallel, the human gut microbiota plays a crucial role in host health, while also being influenced by extrinsic elements (Thursby and Juge, 2017; Visconti et al., 2019; Gacesa et al., 2022). As the gut microbiome is known to promote biotransformation of xenobiotics and other metabolites, many environmental pollutants, including industrial and agricultural chemicals and even heavy metals, have been observed to be metabolized by the gut microbiota (Rosenfeld, 2017; Tsiaoussis et al., 2019; Visconti et al., 2019; Yuan et al., 2019; Sharma et al., 2023). Hence, environmental xenobiotics may directly influence the composition of the gut microbiome, as well as indirectly affecting the production of bacterial metabolites (Claus et al., 2016; Rosenfeld, 2017; Rinninella et al., 2019; Tsiaoussis et al., 2019; Visconti et al., 2019; Sharma et al., 2023). Understanding the effects of occupational pesticide exposure on the gut microbiome can provide valuable insights into the mechanisms that underlie the impact of pesticide exposure on host health.

Despite the advances of the modern multi-omics era, which have allowed for large-scale and deeper characterization of the human gut microbiome and biomonitoring of the exposome, current technological, analytical and inferential barriers continue to limit our ability to fully capture the complexity of these factors and its impact on human health (Sillé et al., 2020; Vermeulen et al., 2020; Chung et al., 2021; Vermeulen, 2022). To date, most pesticide-related studies have been compound-, dosage-, time-and setting-specific, which greatly narrows understanding of the topic (Peters et al., 2021). Although there is evidence that the gut microbiome plays an important role in mediating the effects of environmental stressors, studies have not yet fully acknowledged its relationship to pesticides, nor have they explored this relationship in the context of different types of exposure. To this end, understanding occupational exposure becomes interesting given its repetitive and simultaneous nature (where workers are often exposed more than once and to more than one type of pesticide). Therefore, in this study, we aimed to investigate the effects of occupational pesticide exposure on human gut microbiome composition.

## 2. Materials and methods

### 2.1. Cohort description

Lifelines is a multi-disciplinary prospective population-based cohort study examining, in a unique three-generation design, the health and health-related behaviors of 167,729 people living in the North of the Netherlands. It employs a broad range of investigative procedures in assessing the biomedical, socio-demographic, behavioral, physical and psychological factors that contribute to the health and disease of the general population, with a special focus on multi-morbidity and complex genetics. For the purposes of this study, we used data available for a subpopulation of the Lifelines cohort, the Dutch Microbiome Project (DMP) (Gacesa et al., 2022). The DMP cohort is focused on establishing gut microbiome patterns in health and disease, and it includes additional questionnaires and biological samples collected between 2015 and 2016 alongside the Lifelines baseline data collection.

Within the DMP, we focused our analysis on 7,198 individuals for whom we have data on anthropometrics, antibiotic use, employment details, home urbanicity levels and occupational codes according to the International Standard Classification of Occupation (ISCO). In addition, all selected DMP participants also provided fecal samples, collected between 2015 and 2016.

### 2.2. Metagenomic sequencing and profiling microbiome composition

Gut microbiome data was obtained from fecal samples through whole-genome shotgun sequencing. Samples were collected by the participants at home and frozen immediately. Upon arrival at the Lifelines biorepository, samples were stored at-80oC until DNA extraction. Information on the season of sample collection was also recorded and used in our analysis. The gut microbiome composition was characterized as previously described in Gacesa et al. (2022). In short, microbial DNA isolation was performed using the QIAamp Fast DNA Stool Mini Kit (Qiagen), according to the manufacturer’s instructions. Shotgun metagenomic sequencing was done at Novogene, using the Illumina HiSeq 2000 technology, and samples followed previously described preparation protocols (Gacesa et al., 2022). Metagenomes were profiled following the data analysis steps of the DMP (Gacesa et al., 2022), 1000IBD (Imhann et al., 2019) and Lifelines-DEEP (Tigchelaar et al., 2015) cohorts. In short, metagenomic profiles were filtered from human reads using the KneadData (v.0.10.0) tool. Taxonomic composition was obtained using MetaPhlAn3 (v.3.0.1) (Beghini et al., 2021). Lastly, samples with total read depth < 10 million and/or percentages of eukaryotic or viral abundance ≥25% were excluded.

Microbial alpha diversity was calculated at species-level using Shannon and Simpson indexes from R package “vegan.” To account for each participant’s unique gut microbiome composition, we focused our taxonomic association analyses on bacteria present in at least 20 individuals from the group of participants with exposure estimates to any class of pesticides, which resulted in selection of 168 bacterial species.

### 2.3. Pesticide exposure assessment

To derive estimates of cumulative and categorical occupational exposure to different classes of pesticides, we utilized data from work-related questionnaires, job codes and job-exposure matrices (JEMs). The job codes were retrieved based on the ISCO, using a Computer-Assisted Structured Coding Tool (CASCOT), following the process of Statistics Netherlands (CBS, 2008). These job codes provide a classification system for employment based on the tasks and duties undertaken during work. These codes were combined with the ALOHA+ JEM (Matheson et al., 2005; Skorge et al., 2009) to estimate the levels of exposure to different pesticide classes per job classification. The ALOHA+ estimates are based on the intensity and probability of exposure and yield a semi-quantitative ordinal scale that corresponds to three different levels: no exposure, low exposure and high exposure, numerically represented by a 0–1-2 scale. We obtained exposure estimates for herbicides, insecticides and fungicides and a more general class of pesticides we refer to as “general pesticides.”

For the majority of our population (66%), ISCO codes were retrieved based on employment information collected concomitantly with fecal collection. For participants for whom concurrent information was not available (34%), ISCO codes were retrieved from the baseline questionnaire data. Additional employment information, such as change in unemployment status and duration of employment/unemployment were used to check whether participants had any data discrepancies between both timepoints. If so, additional inspection of the data was made to confirm whether there were no inconsistencies between pesticide exposure estimates for each participant. Lastly, the participant’s ISCO codes, with coding from the 2008 data release, and the ALOHA+ JEM, with ISCO job coding from 1988, were linked via the publicly available correspondence table between distinct ISCO data releases (ISCO-08 and ISCO-88) (ISCO, 2008). Linkage between job codes with distinct pesticide exposure estimates, i.e., ISCO-88 codes that were mapped to multiple ISCO-08 codes that contained distinctive exposure estimates (or vice versa), were analyzed separately and final codes were selected based on similarity of job tasks (Koeman et al., 2013) (Supplementary Table S1).

We calculated cumulative exposure estimates by linking self-reported length of employment with the ALOHA+ estimates (Matheson et al., 2005; van der Mark et al., 2015). Employment questionnaires had categorical answers on length of employment (“Less than 1 year,” “Between 1 and 5 years,” “Between 6 and 10 years” and “More than 10 years”). To quantify these variables numerically, we assigned each category its corresponding mean value (0.5, 3, 7.5 and 15 years). Finally, we multiplied the length of employment by the weighted numeric values of the initial exposure assessment, with 4 indicating high exposure and 1 indicating low exposure, in line with methods used in previous reports (Matheson et al., 2005). In cases where participants were exposed to at least one class of pesticide, at any level, and there was no information regarding their length of employment (24% of the exposed group), we assigned these participants the mean numeric length of employment among the exposed group (11.6 years).

### 2.4. Statistical analysis

We used covariate-adjusted models to investigate the association between occupational exposure to pesticides and microbial alpha and beta diversity as well as to the abundance of microbial species. The models included adjustment for age, sex, body mass index (BMI), season of fecal sampling, mean Bristol Stool score, DNA concentration of the sample and antibiotic use in the past 3 months (Table 1). Comparisons between the results from the adjusted and unadjusted regressions demonstrate that adjusting our model for the covariates increased the power for discovery in the statistical modeling and led to only minor changes in effect estimates. Associations of pesticide classes to alpha diversity and species abundance were calculated using linear regression. For regressions with categorical exposure estimates, an overall assessment of the statistical significance for all categories was produced using ANOVA. Associations to beta diversity (represented as Aitchison dissimilarity) were calculated using PERMANOVA (adonis2 function from R package “vegan”). Prior to association analysis, the abundances of microbial species were transformed using centered log-ratio transformation (adding pseudo count equal to ½ of the smallest non-zero abundance in the dataset, similar to the procedure implemented in the R package “microbiome”) to properly normalize skewed distributions and deal with the compositional nature of the data. For the cumulative exposure estimates, we used inverse-rank sum transformations. All statistical test results were adjusted for multiple testing using the Benjamini-Hochberg procedure.

**Table 1:** Distribution of population characteristics according to exposure subgroups.

	Not exposed	Exposed^*^
	*n* = 6,899	*n* = 299
Sex (%)		
*Males*	2,737 (39.7)	207 (69.2)
*Females*	4,162 (60.3)	92 (30.8)
Age (Mean, SD)	50.7 (11.5)	50.9 (12.3)
BMI (Mean, SD)	26.0 (4.1)	26.0 (4.6)
Season of fecal sampling (%)		
*Summer*	1,610 (24.8)	57 (21.0)
*Fall*	1,505 (23.2)	66 (24.4)
*Winter*	1,643 (25.3)	77 (28.4)
*Spring*	1724 (26.6)	71 (26.2)
Bristol Stool Chart^**^ (Mean, SD)	3.8 (0.9)	3.9 (0.9)
DNA concentration (ng/μL) (Mean, SD)	54.3 (33.5)	54.3 (31.3)
DNA extracted (μg/μL) (Mean, SD)	217.09 (134.03)	217.29 (125.17)
Antibiotic use in past 3 months (%)	426 (7.4)	16 (6.9)

^*^The exposure subgroup was created by selecting individuals in the population who presented exposure estimates to at least one level of exposure in any pesticide class included in our analysis. ^**^As bowel transit time may directly interfere with the composition of the gut microbiome observed in fecal samples, we included the weekly average of the Bristol Stool Chart in our analysis.

## 3. Results

### 3.1. Occupational pesticide exposure in the DMP population

**Figure 1:**
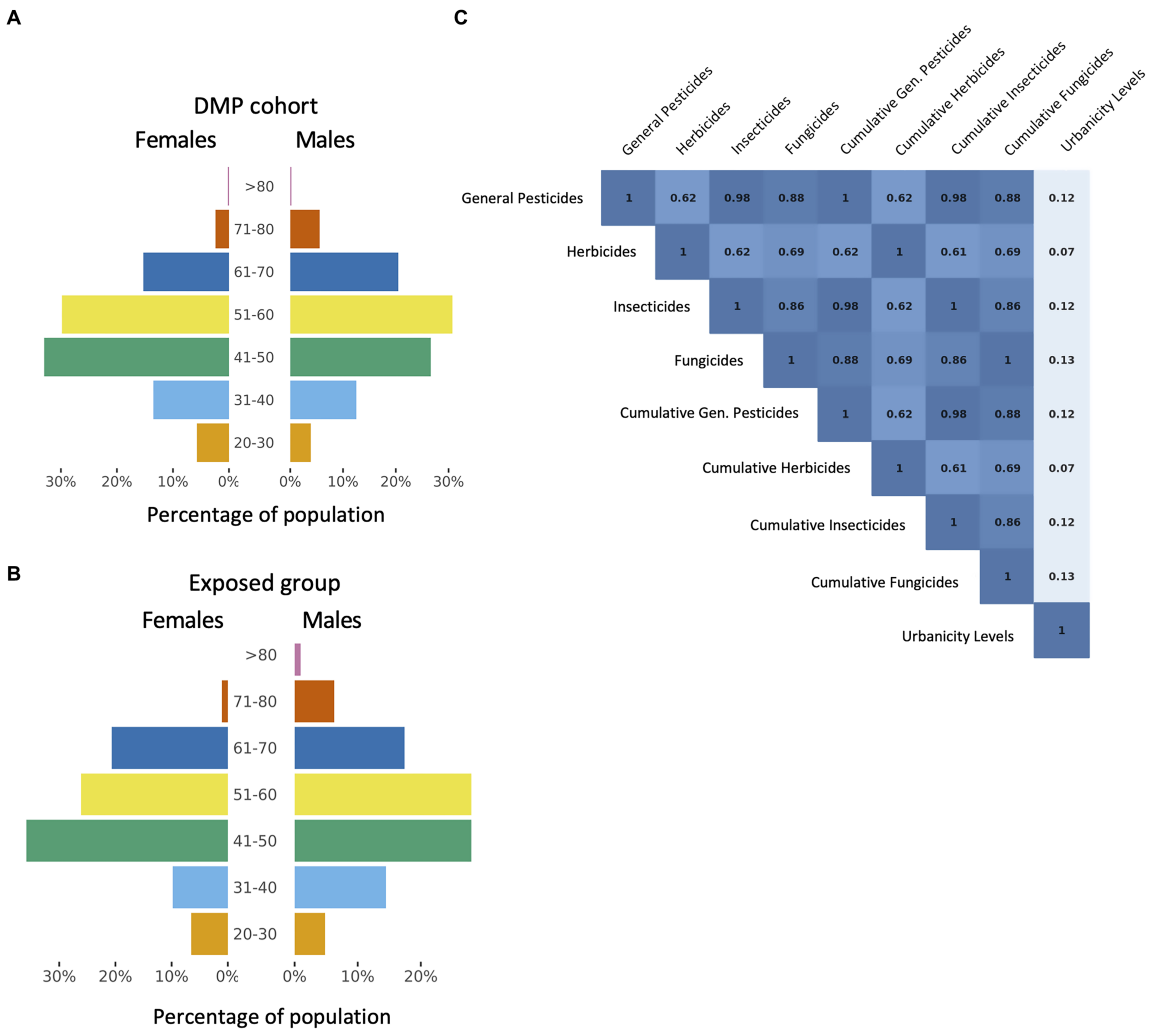
Profile of pesticide exposures. **(A)** Overall distribution of age and sex in the study population shows relatively normal distributions, with higher proportion of females compared to males. **(B)** Distribution of age and sex of participants who show exposure estimates to at least one pesticide class. The proportion of males is higher than in **(A)** but there is a similar age distribution to that of the general population. **(C)** Spearman correlation values between all exposure estimates included in this study and residential urbanicity levels show that exposures often occurred concomitantly across different pesticide classes and are not associated with urban density.

We analyzed data from 7,198 participants of the DMP cohort who reported being employed in at least one timepoint of the DMP data collection. The mean age of the population was 50.7 years, with ages ranging from 20 to 84 years. The majority of the cohort (59%) is female (Figure 1A). Among the participants in our study, 4.1% (*n* = 299) were assigned a level of exposure to at least one type of pesticide (Table 2).

**Table 2:** Distribution of pesticide exposure across different pesticide classes in the DMP cohort.

	General pesticides (% of total)	Herbicides (% of total)	Insecticides (% of total)	Fungicides (% of total)
Not exposed	6,899 (95.8)	7,084 (98.4)	6,911 (96.0)	6,965 (96.7)
*Ever exposed*				
Low	196 (2.7)	81 (1.1)	187 (2.6)	158 (2.2)
High	103 (1.4)	33 (0.4)	100 (1.4)	75 (1.0)
*Duration (years)*				
<1	13 (0.2)	8 (0.1)	13 (0.2)	10 (0.1)
1–5	38 (0.5)	17 (0.2)	36 (0.5)	28 (0.4)
6–10	33 (0.4)	16 (0.2)	31 (0.4)	22 (0.3)
>10	143 (2.0)	41 (0.6)	138 (1.9)	113 (1.6)
Not reported^*^	72 (1.0)	32 (0.4)	69 (0.9)	60 (0.8)

^*^Participants later assigned to the mean numeric length of employment among the exposed group.

The exposed participants had a similar age distribution to the total population, with a mean age of 50.9 years. A higher proportion of males (69.2%) were estimated to be occupationally exposed to pesticides compared to females in the same category (30.8%) (Fischer’s exact test, *p* < 2.2×10^−16^, Odds Ratio = 3.42) (Figure 1B). Occupational exposure to general pesticides, herbicides, insecticides or fungicides was reported in a total of 19 distinct job classifications. Of our exposed group, 37% (*n* = 111) were dairy and livestock producers, 15% (*n* = 46) were freight handlers, 13% (*n* = 39) did general garden-based labor, 9% (*n* = 28) were mixed crop and animal producers, 5% (*n* = 16) were field crop and vegetable growers and 3% (*n* = 11) were mobile farm and forestry plant operators, among other less common occupations.

Overall, low pesticide exposure estimates were more prevalent compared to high exposure estimates, across all pesticide classes (Table 2). Apart from herbicides, which displayed a smaller percentage of exposed individuals (only 1.5% exposed), all other pesticide types had similar exposure rates across the population (3.2% for fungicides, 3.9% for insecticides and 4.1% for general pesticides), and these exposures were often concurrent (3.0% exposed to all three types), resulting in high correlation between these pesticide classes (R_min_ = 0.86 and R_max_ = 0.98) (Figure 1C).

Additionally, data on length of employment was available for 79.8% of the total population. The majority of the participants (53.6%) reported working for the same employer for over 6 years, while only 26.2% indicated being employed for a shorter period. A similar trend was observed when looking at the employment profile across the exposed individuals, where 58.9% declared employment for more than 6 years. The average length of employment within the general cohort was 9.7 years, whereas this was 11.1 years for the exposed group. These observations suggest that the individuals who are occupationally exposed to pesticides had long and potentially repetitive exposure profiles.

### 3.2. Occupational pesticide exposure is not correlated to urbanicity levels

Previous reports have demonstrated that other exposome factors, such as geographical location, socio-economic status and urban and farm-related environments are associated with changes in the human gut microbiome (Segata, 2015; Tasnim et al., 2017; Hildebrand et al., 2021; Gacesa et al., 2022; Valles-Colomer et al., 2023). Because occupational pesticide exposures occur most often in farm, agricultural and garden-related jobs, which are often characteristic of a less urban environment, we sought to clarify whether the occupational pesticide exposure estimates were correlated with the urban density of participant’s residences.

For the DMP population, urbanization levels were determined based on the home address at the time of fecal collection, as described in Gacesa et al. (2022), where the surrounding urban density was represented by a categorical scale from 1 (very urban, ≥2,500 addresses registered per km^2^) to 5 (very rural, ≤500 addresses per km^2^). To investigate the relationship between urbanicity levels and pesticide exposure, we calculated Spearman correlation coefficients between the density scale and all exposure estimates for all pesticide classes for both categorical levels and cumulative estimates (Figure 1C). While exposed occupations in our cohort were primarily linked to farm-related jobs, the urbanicity levels of their residential environment are not strongly correlated to any of the occupational exposure estimates (max. R_spearman_ = 0.13). This suggests that, in the northern region of the Netherlands, residential urbanicity indices do not have a substantial effect on occupational exposures.

### 3.3. Occupational pesticide exposure is linked to changes in the composition of the human gut microbiome

**Figure 2:**
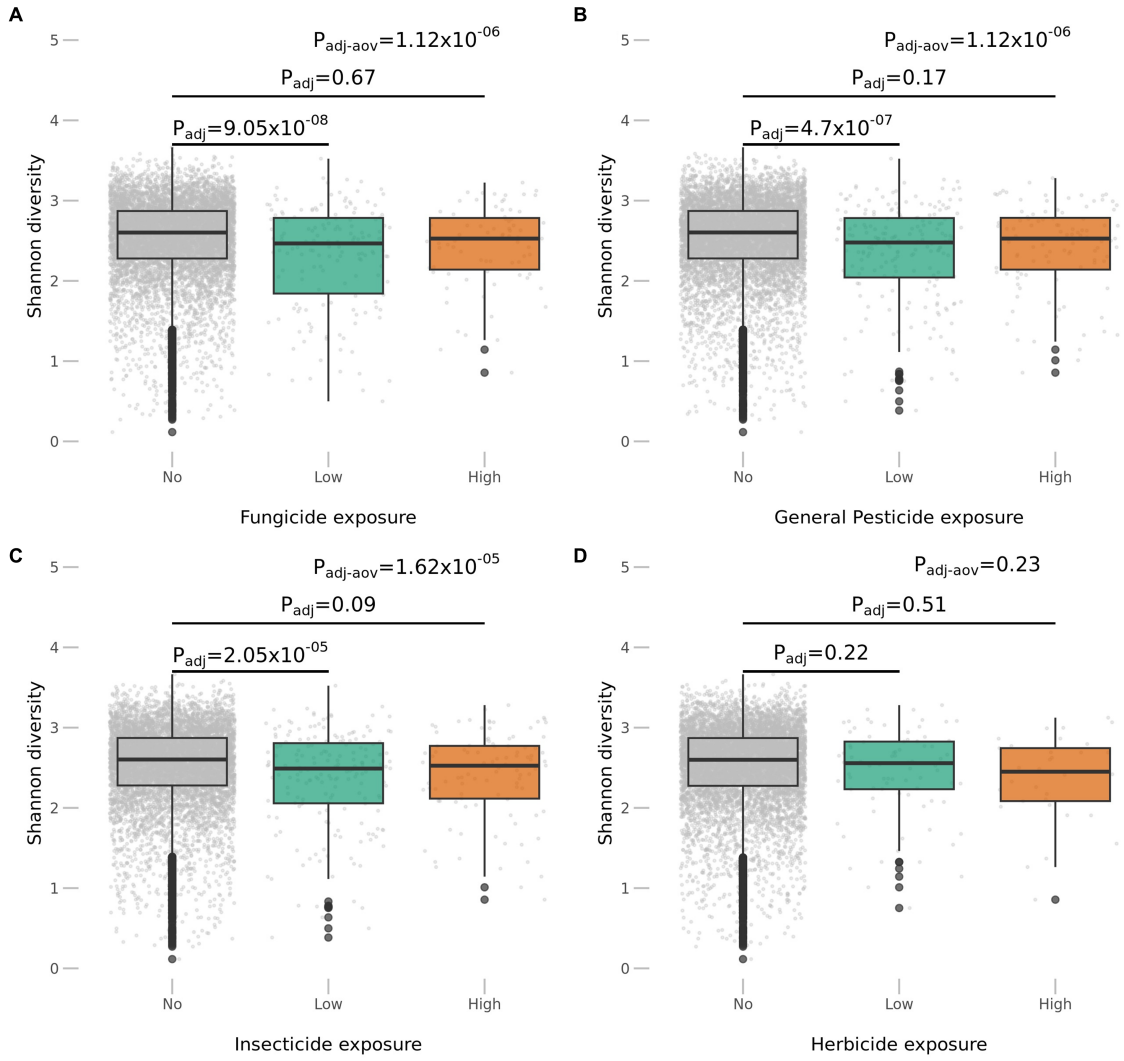
Pesticide exposures are associated with a decrease in alpha diversity. **(A–C)** Individuals exposed to fungicide, pesticide and insecticides show a significant lower diversity. **(D)** Despite a lack of statistical significance, when looking at changes in microbial richness upon herbicide exposure, there is a trend of lower diversity in individuals with estimated exposure.

To investigate the impact of occupational pesticide exposure on the gut microbiome, we compared the alpha diversity of the gut microbiome, measured using the Shannon and Simpson indexes, across the occupational exposure estimates to different pesticide classes and levels. Our results show that Shannon diversity was significantly decreased upon exposure to most pesticide classes. Fungicide exposure was found to be strongly associated with decreased taxonomic diversity (P_adj_ = 1.11×10^−6^), followed by general pesticides (P_adj_ = 1.12×10^−6^) and insecticides (P_adj_ = 2.15×10^−5^) (Figures 2A–C). In contrast, exposure to herbicides did not show any significant associations with changes in microbial alpha diversity (P_adj_ = 0.23) (Figure 2D). Similar results were obtained using the Simpson index (Supplementary Figure S1) The lack of statistical significance for herbicide exposure is most likely due to the much lower number of exposed participants compared to other classes of chemicals, as we still observe a trend of decreased alpha diversity.

We also observed that low exposure estimates were consistently more statistically significant across our associations compared to high exposure estimates. Here, the statistical power may again play a role in the significance of results, as only a small number of participants had high estimates of occupational exposure to pesticides. When looking at cumulative exposure estimates, we observed similar results to the categorical analyses, with exposure to fungicides, general pesticides and insecticides significantly associated with decreased microbial diversity but again no statistically significant association to herbicide exposure (Table 2).

To further analyze the gut microbiome beta diversity across individuals with distinct exposure profiles, we performed PERMANOVA analyses. This allowed us to explore the dissimilarities in the microbial community structure given the exposures to different classes of pesticides. In line with the alpha diversity results, exposures to general pesticides, fungicides and insecticides were significantly associated with a distinct gut microbiome composition (Table 3), while herbicide exposure was not significantly associated with overall microbial composition.

**Table 3:** Associations between alpha and beta diversity and pesticide exposure estimates.

Alpha diversity		Estimate	*T*-value	*p*-val	*p*-val_adj_	*p*-val_aov_	*p*-val_aov-adj_
*Categorical estimates*							
General Pesticide exposure	Low	−0.19	−5.17	2.35×10^−7^	4.70×10^−7^	5.61×10^−7^	1.12×10^−6^
	High	−0.08	−1.56	0.12	0.17		
Herbicide exposure	Low	−0.08	−1.50	0.13	0.22	0.23	0.23
	High	−0.07	−0.82	0.41	0.51		
Insecticide exposure	Low	−0.17	−4.37	1.23×10^−5^	2.05×10^−5^	1.62×10^−5^	2.15×10^−5^
	High	−0.10	−1.82	0.07	0.09		
Fungicide exposure	Low	−0.23	−5.47	4.52×10^−8^	9.05×10^−8^	2.79×10^−07^	1.12×10^−6^
	High	−0.03	−0.62	0.53	0.67		
*Cumulative estimates*							
General Pesticide exposure		−0.06	−4.77	1.84×10^−6^	2.77×10^−6^		
Herbicide exposure		−0.03	−1.71	0.09	0.13		
Insecticide exposure		−0.06	−4.38	1.21×10^−5^	1.81×10^−5^		
Fungicide exposure		−0.07	−4.51	6.51×10^−6^	9.77×10^−6^		

*Beta Diversity*	*R* ^2^	*F*-stats	*p*-val_Permanova_^*^
General Pesticide exposure	5.78×10^−4^	3.94	9.99×10^−4^
Herbicide exposure	2.03×10^−4^	1.39	3.10×10^−2^
Insecticide exposure	5.91×10^−4^	4.03	9.99×10^−4^
Fungicide exposure	5.84×10^−4^	3.99	9.99×10^−4^

Alpha diversity was estimated using Shannon index. Beta diversity was retrieved by representations of Aitchison dissimilarity. *PERMANOVA tests were performed using 1,000 permutations.

Our findings provide evidence that occupational pesticide exposure is associated with changes in the diversity and composition of the gut microbiome. We demonstrate that the impact of such exposures may follow different patterns according to distinct classes of pesticide exposure.

### 3.4. Pesticide exposure impacts the abundance of specific microbial taxa in the human gut

To inspect the effect of these exposures on specific gut microbial features, we explored associations between the different types of pesticide exposures and the relative abundance of the 168 bacterial species that passed our selection criteria. Our analysis included both the categorical levels of exposure and cumulative exposure estimates, and all statistical tests were corrected for age, sex, BMI, season of sampling, mean Bristol Stool score, DNA concentration of the sample and antibiotic use in the past 3 months (Table 1). Rather than relying solely on statistical associations for each level of exposure, we compared the difference between all categories of exposure for the same pesticide class using an additional ANOVA test.

In total, 39 bacterial species were significantly associated with exposure to at least one class of pesticide (Supplementary Table S2). Many of the significant associations between specific taxa and pesticide exposure estimates were consistent across the different classes of pesticides, with 26 bacterial species displaying significant associations with general pesticides, fungicides and insecticides.

**Figure 3:**
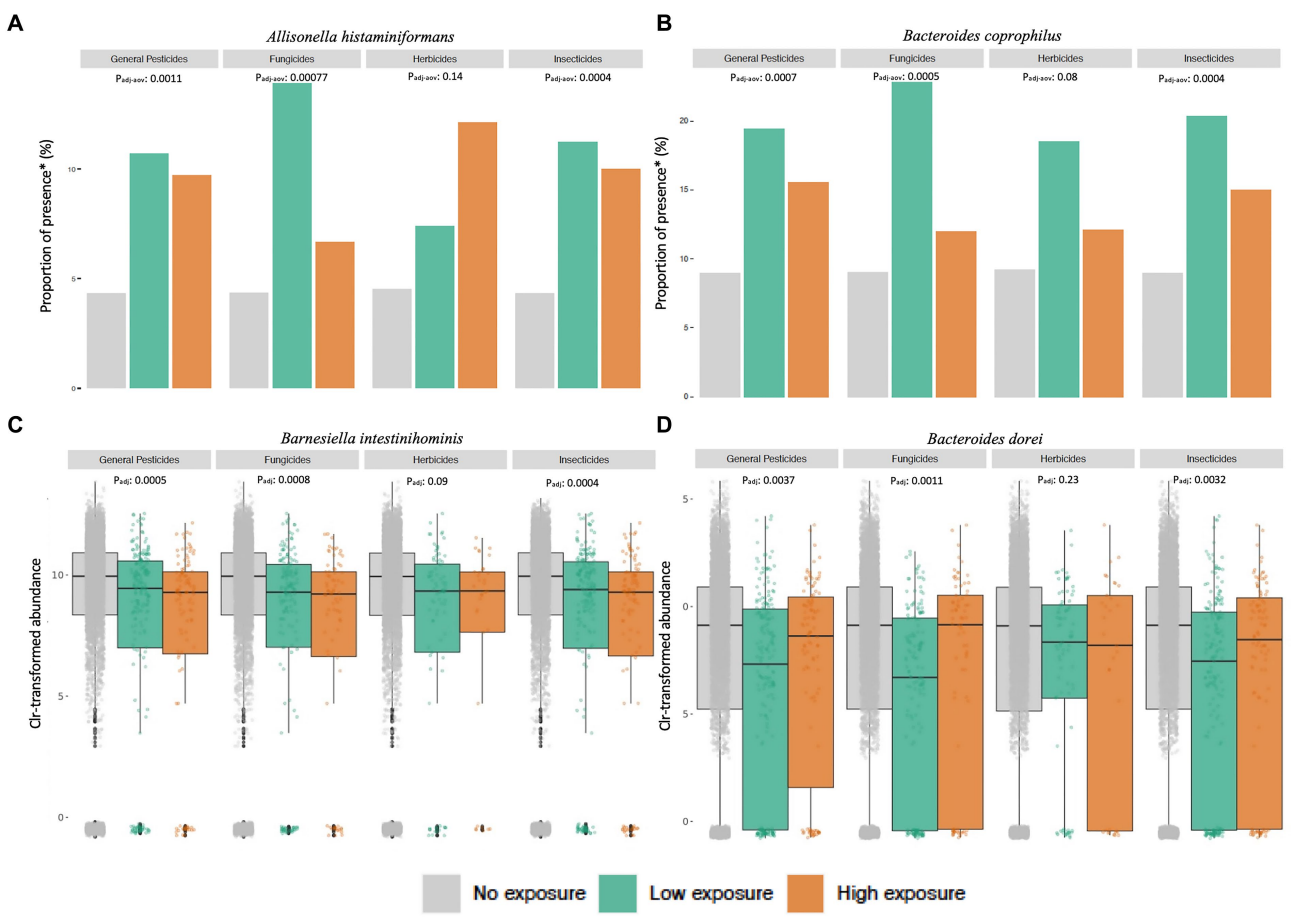
Modulations in microbial taxa upon pesticide exposures. Significant associations between exposures and microbial abundance can be observed across a considerable portion of the bacterial taxa included in this study. Higher proportions of presence of **(A)** *Allisonella histaminiformans* and **(B)** *Bacteroides coprophilus* can be observed at different levels of exposures. There are also changes in microbial abundance with exposure levels, particularly a decrease in **(C)** *Barnesiella intestinihominis* and **(D)** *Bacteroides dorei* across exposure levels and pesticide types. *Proportions of presence are calculated separately for each level of exposure.

We observed that exposure to all types of pesticides was significantly associated with the increased presence of *Allisonella histaminiformans*, *Bacteroides coprophilus*, *Mitsuokella multacida* and *Parabacteroides* sp. *CAG 409* (Figures 3A,B). Conversely, the abundance of *Barnesiella intestinihominis* was decreased for exposures of all types (Figure 3C). Most of the impact of exposures was related to changes in the presence or absence of low-abundance bacteria like *Acidaminococcus fermentans* and *Megasphaera elsdenii*, but less commonly it was related to changes in the abundance of more abundant bacteria, such as decrease of *Bacteroides dorei, Akkermansia muciniphila* and *Alistipes finegoldii* (Figure 3D).

Of note, in the associations with specific taxa, we could see effects of herbicide exposure. However, from our analyses, only two bacterial species, *Phascolarctobacterium faecium* and *Roseburia faecis*, were significantly associated with herbicide exposure alone, and these signals were only marginally significant (P_aov-adj_ = 0.04 and 0.03, respectively). Additionally, these and other associations, such as with *Allisonella histaminiformans* and *Barnesiella intestinihominis,* only remained statistically significant after multiple-testing correction in tests with cumulative exposure to herbicides.

## 4. Discussion

Numerous studies have reported associations between environmental pollutants and changes in the microbiome in a wide range of settings, from soil to humans (Rosenfeld, 2017; Iszatt et al., 2019; Yuan et al., 2019; Giambò et al., 2021; Daisley et al., 2022; Naspolini et al., 2022). However, properly accounting for the complexity of such environmental factors can be tricky. To date, most analyses are compound-, setting-or exposure-specific, or derived from biomonitoring, which is dependent on proper detection methods and databases (Stanfield et al., 2022). Inspired by the limited understanding of the impact of these exposures on the gut microbiome, we performed a more integrative investigation of the impact of occupational pesticide exposures on the gut microbiome.

We focused on a subset of 7,198 participants from the DMP to investigate the link between the gut microbiome profile and occupational pesticide exposures. To assess occupational exposures, we examined four distinct pesticide classes: fungicides, herbicides, insecticides and a “general pesticides” class, while exploring categorical (No, Low and High) and cumulative (weighted years) exposure estimates. We found that approximately 4% of our DMP population were exposed to at least one class of pesticides at their employment. The proportion of male participants was higher in the exposed group, and participants were employed for an average of 11.1 years, showing a predominance of chronic exposure profiles.

Our results show that exposures to insecticides, fungicides and general pesticides are consistently associated with changes in the gut microbiome, as shown by associations with decreased alpha diversity and significant differences in microbial community structure. Further, we identify changes in the abundances of 39 different bacterial taxa upon exposure to the different types of pesticides included in this study.

A decrease in gut microbiome alpha diversity, as reported in this study, has been previously linked to adverse health outcomes (Rinninella et al., 2019; Visconti et al., 2019; Manor et al., 2020). In addition, exposed participants showed a decrease in abundance of taxa such as *Bacteroides dorei* and *Alistipes finegoldii* that have been previously linked to diseases and disturbances of the human gut microenvironment (Davis-Richardson et al., 2014; Parker et al., 2020). We also observe an increased presence of low-abundance bacteria such as *Allisonella histaminiformans*, *Acidaminococcus fermentans*, *Megasphaera elsdenii* and *Mitsuokella multacida*. Little is known about the exact role of these bacteria in the human gut, but *Allisonella histaminiformans* is known to produce histamine, which can induce mucus production (Garner et al., 2002). We also observed interesting associations with butyrate producers, such as *Mitsuokella multacida* and *Akkermansia muciniphila*, but the functional consequences of each of the reported associations should be further explored in future studies. Further, we found that herbicide exposure showed less significant associations with microbial taxa, diversity and composition compared to other pesticide classes, but it is not possible to determine based on our results alone whether this effect is due to lack of statistical power, compound-specificity, increased toxicity due to the combined use of the other pesticide classes or another unknown factor.

Although occupational exposure estimates are not direct biological measures for compound uptake, the significant proportion and consistency of statistically relevant associations with pesticide exposure observed in this study demonstrates the extensive impact of these exposures on the human gut environment. Still, functional studies are needed to confirm any hypothesis. We also acknowledge the limitations of using JEMs to capture the complexity of lifetime occupational exposures by deriving estimations from probability, intensity and length of employment (Stewart and Stenzel, 2000; Matheson et al., 2005). However, JEMs have been reported to be suitable methods for assessment of exposure estimates in general population designs that also allow for estimation of longer-term exposure (Allen et al., 2006; Dalvie et al., 2009; Carles et al., 2017; Ohlander et al., 2020). On this matter, studies have observed good agreement between JEMs and biomonitoring findings (Carles et al., 2017; Ohlander et al., 2020). An advantage of this methodological approach is that it predominantly produces Berkson-type errors, rather than classical error-structure, which can affect precision, but given its nature, does not introduce bias in effect estimates (Armstrong, 1998; Heid et al., 2004; Faruque et al., 2021). Another source of uncertainty may be our inability to determine and distinguish whether we are looking at acute or chronic perturbations of the gut microbiome, as we are using cross-sectional microbiome data. Yet, given the methodological difficulties of the field, we report reasonable evidence for the impact of occupational pesticide exposure on the human gut microbiome.

Overall, our study highlights the potential effect of occupational pesticide exposure on the human gut microbiome and the need for further studies to elucidate the mechanisms involved. Additional investigations should expand our current understanding of the impact of these microbial changes on host health. Finally, our findings underscore the importance of biomonitoring and of further regulating pesticide exposures.

## Data availability statement

The datasets presented in this study can be found in online repositories. The names of the repository/repositories and accession number(s) can be found at: https://ega-archive.org/studies/EGAS00001005027.

## Author contributions

MG, RV, AZ, VL, and AK contributed to the conception and design of the study. MG, AH, and VL linked the datasets and the Job Exposure Matrix. MG, AF-P, AH, VL, RV, AZ, and AK contributed to the analyses and interpretation of data. MG wrote the first draft of the manuscript. All authors contributed to the article and approved the submitted version.

## Funding

This research received funding from the EXPOSOME-NL Gravitation program of the Dutch Ministry of Education, Culture, and Science and the Netherlands Organization for Scientific Research (NWO grant number 024.004.017). RW is supported by the collaborative TIMID project (LSHM18057-SGF) financed by the PPP allowance made available by Top Sector Life Sciences & Health to Samenwerkende Gezondheidsfondsen (SGF) to stimulate public–private partnerships and co-financed by health foundations that are part of the SGF. AZ is supported by the European Research Council (ERC) Starting Grant 715772, the Netherlands Organization for Scientific Research (NWO) VIDI grant 016.178.056 and NWO Gravitation grant ExposomeNL 024.004.017. JF is supported by NWO Gravitation grant Netherlands Organ-on-Chip Initiative 024.003.001, ERC Consolidator grant 101001678 and NWO VICI grant VI.C.202.022. JF and AZ are supported by the Netherlands Heart Foundation CVON grant 2018–27. RW is supported by the Seerave Foundation and the Dutch Digestive Foundation (16–14). CW is supported by NWO Gravitation grant 024.003.001 and NWO Spinoza Prize SPI 92–266.

## Conflict of interest

The authors declare that the research was conducted in the absence of any commercial or financial relationships that could be construed as a potential conflict of interest.

## Publisher’s note

All claims expressed in this article are solely those of the authors and do not necessarily represent those of their affiliated organizations, or those of the publisher, the editors and the reviewers. Any product that may be evaluated in this article, or claim that may be made by its manufacturer, is not guaranteed or endorsed by the publisher.

## Supplementary material

The Supplementary material for this article can be found online at: https://www.frontiersin.org/articles/10.3389/fmicb.2023.1223120/full#supplementary-material
